# Effectiveness of Traditional Chinese Acupuncture versus Sham Acupuncture: a Systematic Review

**DOI:** 10.1590/1518-8345.0647.2762

**Published:** 2016-08-15

**Authors:** Luís Carlos, Lóris Aparecida Prado da Cruz, Vanessa Cristina Leopoldo, Fabrício Ribeiro de Campos, Ana Maria de Almeida, Renata Cristina de Campos Pereira Silveira

**Affiliations:** 1 Doctoral Student, Escola de Enfermagem de Ribeirão Preto, Universidade de São Paulo, PAHO/WHO Collaborating Centre for Nursing Research Development, Ribeirão Preto, SP, Brazil.; 2 Master's Student, Escola de Enfermagem de Ribeirão Preto, Universidade de São Paulo, PAHO/WHO Collaborating Centre for Nursing Research Development, Ribeirão Preto, SP, Brazil.; 3 Master's Student, Escola de Enfermagem de Ribeirão Preto, Universidade de São Paulo, PAHO/WHO Collaborating Centre for Nursing Research Development, Ribeirão Preto, SP, Brazil. RN, Hospital das Clínicas, Faculdade de Medicina de Ribeirão Preto, Universidade de São Paulo, Ribeirão Preto, SP, Brazil.; 4 Master's Student, Escola de Enfermagem de Ribeirão Preto, Universidade de São Paulo, PAHO/WHO Collaborating Centre for Nursing Research Development, Ribeirão Preto, SP, Brazil. RN, Fundação Santa Casa de Misericórdia de Franca, Franca, SP, Brazil.; 5 PhD, Associate Professor, Escola de Enfermagem de Ribeirão Preto, Universidade de São Paulo, PAHO/WHO Collaborating Centre for Nursing Research Development, Ribeirão Preto, SP, Brazil.; 6 PhD, Professor, Escola de Enfermagem de Ribeirão Preto, Universidade de São Paulo, PAHO/WHO Collaborating Centre for Nursing Research Development, Ribeirão Preto, SP, Brazil.

**Keywords:** Breast Neoplasm, Acupuncture, Placebos, Hot Flashes, Menopause

## Abstract

**Objective:**

to identify and synthesize the evidence from randomized clinical trials that tested the effectiveness of traditional Chinese acupuncture in relation to sham acupuncture for the treatment of hot flashes in menopausal women with breast cancer.

**Method:**

systematic review guided by the recommendations of the Cochrane Collaboration. Citations were searched in the following databases: MEDLINE via PubMed, Web of Science, CENTRAL, CINAHL, and LILACS. A combination of the following keywords was used: breast neoplasm, acupuncture, acupuncture therapy, acupuncture points, placebos, sham treatment, hot flashes, hot flushes, menopause, climacteric, and vasomotor symptoms.

**Results:**

a total of 272 studies were identified, five of which were selected and analyzed. Slight superiority of traditional acupuncture compared with sham acupuncture was observed; however, there were no strong statistical associations.

**Conclusions:**

the evidence gathered was not sufficient to affirm the effectiveness of traditional acupuncture compared with sham acupuncture.

## Introduction

Women in menopause experience a number of vasomotor symptoms, among which hot flashes prevail. It is estimated that between 50% and 80% of menopausal women experience hot flashes^1^, and these ratios may vary from 64% to 85% in women with breast cancer^2^. The intensity and duration of hot flash episodes in women vary, and hot flashes generally lead to reduced quality of life because they cause physical discomfort and irritability, disturb sleep, and can lead to depression^1^ ^-^ ^3^.

Despite increases in the survival rate of patients with cancer, there is still a need for research to assess the effect of treatments on their quality of life. The use of complementary therapies to decrease the treatment side effects is being increasingly documented internationally in several populations, especially in menopausal women with breast cancer^4^ ^-^ ^7^.

A growing body of scientific evidence from well-designed studies supports the use of complementary therapies to manage the vasomotor symptoms of menopause, especially hot flashes in women with breast cancer, which is the focus of this review^8^ ^-^ ^11^. Among these practices, the use of acupuncture for the relief of hot flashes has been demonstrated to be potent and complementary to conventional treatment, and it is well-accepted by patients in terms of effectiveness^12^.

Acupuncture is among the most popular forms of complementary medicine^13^ ^-^ ^14^, and its use is related to improvements in psychological symptoms through sympathomimetic pathways^15^ ^-^ ^16^. Thus, studies have tested the hypothesis that acupuncture reduces the frequency of hot flashes, thus improving the quality of life of menopausal women with cancer ^10^ ^,^ ^17^.

Traditional Chinese acupuncture (TCA) is used as a complement to conventional treatment for various pathological conditions. It aims to relieve symptoms via the reorganization of the body's energy, with the goal of self-cure^18^ ^-^ ^19^. Sham acupuncture (SA), also called placebo, may be considered a fake intervention, as it is performed off the acupuncture points established by TCA^20^ ^-^ ^21^.

The lack of studies with acceptable controls that mimic all aspects of the tested intervention has been the main methodological problem in studies that use acupuncture as a treatment. In the past, the lack of an adequate simulated procedure (sham treatment) led researchers to compare real acupuncture with a wide range of interventions^22^ ^-^ ^24^. Although a systematic review performed in 2009^10^ evaluated the efficacy of acupuncture as a treatment option for hot flashes in patients with breast cancer, the main focus of this review was not specifically to compare TCA to SA. Instead, the authors reviewed many clinical trials comparing TCA with various types of treatment, including SA; active treatments, such as hormone therapy, relaxation, and antidepressants; and no treatment. The authors concluded that the evidence that acupuncture is an effective treatment for the relief of hot flashes in patients with breast cancer was not convincing, mainly because of systematic errors in the studies reviewed. 

The authors hypothesized that the fact that TCA did not produce statistically superior results compared with SA, regardless of the technique used, was because the TCA was either ineffective or was not administered correctly or because SA is also an effective treatment. From this perspective, we sought to update and present the state of the art on this particular issue, as SA has been a reliable method showing promising results for some morbidities^25^ ^-^ ^26^.

The rationale for the present study is based on the high prevalence of hot flashes in menopausal women with breast cancer. This symptom is associated with a worse prognosis^1^ ^-^ ^3^, including a low survival rate, reduced adherence to treatment, and diminished overall quality of life. Thus, managing this symptom is an important aspect of nursing practice in oncology. Assessing and treating symptoms related to cancer in terms of patient survival, adherence to treatment, and quality of life during and after treatment is currently among the research pillars of clinical oncology and is a priority in oncology nursing research^27^. Given the importance of the subject, the aim of the present study was to identify and synthesize evidence from randomized controlled clinical trials that tested the effectiveness of TCA in relation to SA for the treatment of hot flashes in menopausal women with breast cancer. 

We believe that grouping, updating, and synthesizing the available evidence on this subject can not only guide further research but also support health professionals' decisions regarding the use of complementary therapies for cancer patients. The goal is to enable the safe and evidence-based use of these therapies to demystify them. 

## Method

The study is a systematic review of the literature, guided by recommendations from the Cochrane Collaboration^28^. The question of the systematic review, which was based on the patient, intervention, comparison, outcome (PICO)^29^ ^)^ strategy, was *"Is traditional Chinese acupuncture more effective than sham acupuncture for the relief of hot flashes in menopausal women with breast cancer?"*

The inclusion criteria were as follows: randomized clinical trials (RCT) published in full up to the present time (July 2014) in English, Portuguese, or Spanish that included adult women (≥18 years old) with breast cancer and in menopause experiencing hot flashes and that analyzed the effectiveness of traditional Chinese *versus* sham acupuncture to treat this vasomotor symptom. Studies conducted with animals and publications such as literature reviews, dissertations, theses, editorials, and clinical guidelines were excluded. 

### Search strategy

We searched five electronic databases: the Medical Literature Analysis and Retrieval System Online via PubMed, Web of Science, the Cochrane Central Register of Controlled Trials Database (CENTRAL), the Cumulative Index of Nursing and Allied Health Literature (CINAHL), and Literature in the Health Sciences in Latin America and the Caribbean (LILACS). The search strategy considered search terms related to the study population (P), intervention (I), comparison with placebo (C), outcome (O), and study design (RCT). We selected keywords from the controlled vocabularies of each database as well as non-controlled keywords, which were combined within each term set with the Boolean connectors AND and OR. The main keywords adopted in the search strategy for the primary studies were *Breast Neoplasm, Acupuncture, Acupuncture Therapy, Acupuncture Points, Placebos, Sham Treatment, Hot Flashes, Hot Flushes, Menopause, Climacteric, Vasomotor Symptoms,* and *Vasomotor Symptoms Menopause,* combined with the Boolean operators AND and OR. To locate the RCTs, we added a filter after the PICO search strategy that included the following terms: AND *Clinical Trial* OR *Controlled Clinical Trial* OR *Randomized Controlled Trial*. The search was performed at the end of July 2014, and 272 publications were initially found. 

### Study selection 

To select the studies, two reviewers independently screened the titles and abstracts of the identified publications. In cases of doubt or disagreement, a third reviewer was asked to decide whether to include the study. The agreement rate between the reviewers was 96%. 

### Methodological quality appraisal of the included studies 

For the methodological quality appraisal of the included studies, we used the Jadad scale^30^, which allows a classification of the quality of the evidence from RCTs and has been described in the literature as a reliable and widely used tool to appraise the quality of clinical trials. This scale appraises and scores five specific topics: 1. Was the study described as randomized?, 2. Was the randomization procedure appropriate?, 3. Was the study described as double-blinded?, 4. Was the concealment method appropriate? and 5. Was there a description of the exclusion criteria and the drop-out rate?. The final score of the Jadad scale ranges from 0 to 5. Studies that score < 3 are classified as low quality, and studies that score ≥ 3 are classified as high quality^30^.

The studies were also appraised regarding the risk of bias, considering random sequence generation; the allocation concealment; the blinding of subjects, health care providers, and outcome evaluators; incomplete outcome data; selective reporting; and other sources of bias^28^ ^,^ ^31^. Studies with a low risk of bias are considered unlikely to have serious problems with the reliability of their results. An uncertain risk of bias raises questions regarding the reliability of the study results, and a high risk of bias seriously weaknesses the reliability of the results^31^.

### Data extraction and analysis

For data extraction, we used a form that was designed for the present study, which considered the instructions provided by the Cochrane Collaboration^28^ ^)^ regarding content and structure. This pre-defined form included the following information: study identification (title, journal, publication year, volume, number, and authors), objectives, and method (randomization method, concealment, number of patients randomized, description of loss to follow-up rates, inclusion and exclusion criteria, measurement of hot flashes and clinical characteristics, intervention in the experimental and control groups, data analysis, and outcomes). The data were extracted from each study by two independent reviewers. Next, all of the selected studies were distributed among three reviewers, who appraised the methodological quality of each trial using the Jadad scale^30^. The data extracted from the studies included in this review were analyzed according to their outcomes, and the results are presented in descriptive form.

## Results 

**Figure 1:**
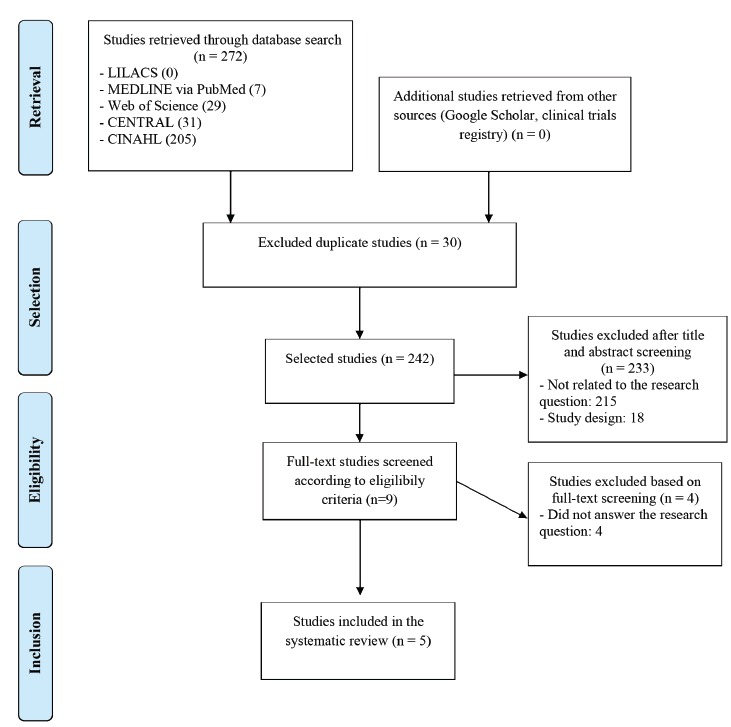
Flowchart of study retrieval, selection, and inclusion.

A total of 272 studies were retrieved from the five databases selected for this study: 205 from CINAHL, 31 from CENTRAL, 29 from Web of Science, 7 from MEDLINE via PubMed, and zero from LILACS. Of these, 242 studies were preselected. After analysis and with 100% agreement among reviewers, only 5 manuscripts met all of the eligibility criteria and answered the proposed research question. Figure 1 shows the flowchart of the selection process for the studies that were included in the present review. The opinion of a third reviewer was requested to obtain consensus on the quality appraisal of the five selected articles. 

Among the included studies, one was conducted in Norway^32^, one in Sweden^33^, one in Denmark^34^ and two in the United States^8^ ^,^ ^35^. Regarding authorship, physicians were predominant in all studies^8^ ^,^ ^32^ ^-^ ^35^. Other professionals who authored the studies included statisticians, physical therapists, physical educators, and pharmacists^8^ ^,^ ^32^ ^-^ ^33^ ^,^ ^35^. 

Regarding the methodological quality of the five RCTs, two were single-blinded^32^ ^-^ ^33^, three were double-blinded^8^ ^,^ ^34^ ^-^ ^35^, and only one was multi-centered^35^. The classification of the evidence level measured according to the Jadad scale^30^ ^)^ showed that all the studies included in the present systematic review were high quality, with one receiving a score of 3^32^, two a score of 4^33^ ^-^ ^34^ and two the maximum score of 5^8^ ^,^ ^35^.

The number of study participants included in the samples varied from 47 to 94, and the women's age varied from 36 to 85 years old. All of the subjects were ambulatory patients. Regarding the treatments, four studies allocated the study subjects into two groups, with one group receiving TCA sessions and the other receiving SA^8^ ^,^ ^32^ ^-^ ^33^ ^,^ ^35^. One trial consisted of three groups, with one undergoing TCA, one group undergoing SA, and a third control group receiving no treatment intervention^34^. 

Treatment length varied from 4 to 12 weeks. One study had a treatment duration of four weeks^8^, two trials had a treatment duration of five weeks^33^ ^-^ ^34^, one study included 10 treatment weeks,^32^ and one included 12 treatment weeks^35^. According to the intervention protocols of the included studies, the number of acupuncture sessions varied from 5 to 15^8^ ^,^ ^32^ ^-^ ^35^.

Regarding the protocols of the interventions, it was observed that in study 1^8^, the needles used in the TCA group were filiform, made of stainless steel, and measured 0.20 x 30 mm (manufactured by SeirinCorp - Shizuoka, Japan). The needles were inserted 0.25 to 0.5 inches into the skin. The acupuncture points were designed and manually manipulated to obtain Qi (vital energy that flows in the meridians), and no electrical stimulus or other interventions were applied. In the SA group, 0.30 × 30 mm Streitberger needles (Asiamed, Pullach, Germany) were used. The needles were applied a few centimeters away from the TCA points (DU14, GB20, BL13, PC7, H6, K7, ST36, and SP6). Rather than penetrating the skin, the needle was retracted after insertion through adhesive tape, which was placed over a supporting plastic ring. This type of fake needle has high reliability and has been used with success in RCTs. The frequency and duration of the intervention in both groups was identical. 

In study 2^32^, the TCA group was treated with disposable 0.3-mm needles inserted from 0.5 to 3.0 cm into the skin. Eight unilateral acupuncture points were used along the TCA meridians (used for cooling the body, toning the yin, and reducing excess heat). The designated acupuncture points were manually manipulated to obtain De Qi, which is numbness, distension, or electrical tingling sensation at the needle insertion sites that may radiate along the corresponding meridian. The target acupuncture points in study 2 were highly innervated areas: LIV3, GB20, LU7, KI3, SP6, REN4, P7, and LIV8. In the SA group, the needles were inserted 2- to 3-mm deep at eight points (four bilateral points) far from the TCA points.

In study 3^33^, the TCA group was treated with eight sterile, disposable, 0.25 × 40-mm needles (Dongbang Acupuncture, Inc.). The needles were inserted to a depth of 5 to 20 mm at defined points until the De Qi threshold was reached. After 10 minutes, the needles were slightly rotated without evoking the needle sensation. The manually manipulated points were Li4, HT6, LR3, and ST36 unilaterally and Sp6 and Ki7 bilaterally. In the SA group, the sham needles were applied 1 cm away from the traditional TCA points without being inserted into a meridian or a different point and without penetrating the skin.

In study 4^34^, the TCA patients received manual manipulation bilaterally for 15-20 minutes at points pre-determined by traditional Chinese medicine. Real points were used along the acupuncture meridians (HC6, KI3, Sp6, and Lr3). The SA group received sham acupuncture at four non-bilateral pre-determined points that were outside the established TCA meridians but in the same region as the real points. The SA intervention used the same type of needles used in the TCA group, but the needles were inserted superficially (1 cm into the skin). This study also included a third group that received no intervention for the purpose of comparison. In study 5^35^, the experimental group received manual TCA in acupoints VC4, CV6, CV12, LI4, MH6, GB34, ST36, KI3 and BL65. In the SA group, the patients received SA with non-penetrating and retractable needles placed in 14 points along the central meridian of the line connecting the real TCA points.

The method used to measure hot flashes in most studies was through field notes^8^ ^,^ ^33^ ^-^ ^35^. One study used the Kupperman index, which assesses menopause symptoms^32^. Two studies used scales to assess the intensity of hot flashes, with one study using a verbal interval four-point Likert scale (no problems, mild, moderate, severe, or extremely severe)^33^ and another study using a visual analogue scale (VAS) ranging from 0 to 10^34^. In one of the studies, other instruments were also used to assess menopause symptoms, including the National Surgical Adjuvant Breast and Bowel Project (NSABP) and the Hot Flash-Related Daily Interference Scale (HFRDI) to assess the effects of hot flashes in everyday life. This same study assessed sleep quality and disturbances using the Pittsburgh Sleep Quality Index (PSQI), depression symptoms using the Center for Epidemiologic Studies Depression Scale (CESD), anxiety using the Hospital Anxiety and Depression Scale (HADS-A), and global quality of life using the European Quality-of-Life Survey (EuroQol)^35^. 

Regarding long-term treatment effectiveness, three studies^8^ ^,^ ^32^ ^,^ ^34^ followed the participants after the completion of the intervention. Among these three studies, one monitored patients up to six weeks after treatment completion^8^, and two studies monitored patients up to 12 weeks after treatment^32^ ^,^ ^34^.

**Figure 2:**
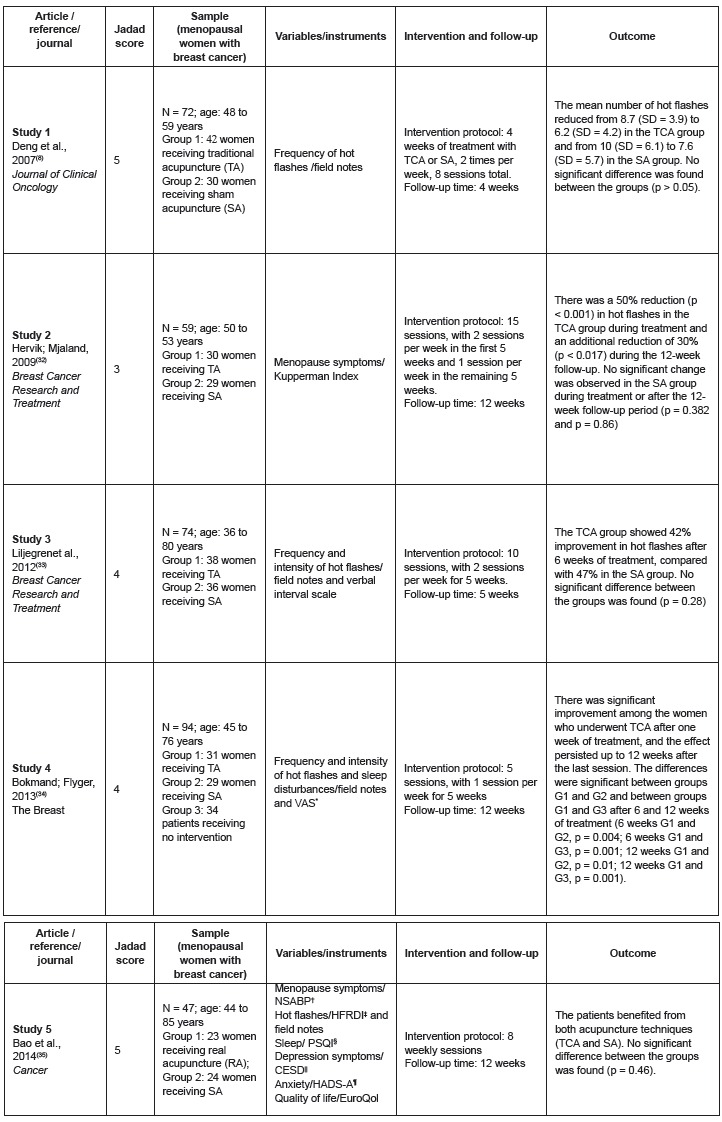
Final and integrated summary of the studies included in the systematic review. 2014

In summary, three studies suggested that TCA is more effective than SA (Figure 2). Study 1^8^ demonstrated that the group receiving TCA experienced 0.8 fewer hot flashes per day than the placebo group receiving SA. However, this association was not statistically significant (CI_95%_= - 0.7 to 2.4; p = 0.30). The results from study 2^32^ ^)^ showed that the number of hot flashes was reduced by 50% (p <0.001) in the group of women receiving TCA, representing an average of 9.5 hot flashes (SD = 4.9) that decreased to 4.7 hot flashes (SD=3.7) during treatment with TCA. No significant change was observed in the group that received SA either during treatment (p = 0.382) or after the 12-week follow-up (p=0.86). In this same study, the difference in response between the TCA group and the SA group was significant during both the treatment and the 12-week follow-up period (p < 0.001). 

*VAS - visual analogue scale; †NSABP - National Surgical Adjuvant Breast and Bowel Project; ‡HFRDI - Hot Flash-Related Daily Interference Scale; §PSQI - Pittsburgh Sleep Quality Index; ||CESD - Center for Epidemiologic Studies Depression Scale; ¶HADS-A - Hospital Anxiety and Depression Scale; EuroQol - European Quality-of-Life survey.

Study 4^34^ found significant associations among the three groups (G1- TCA; G2- SA; G3- no intervention). There was a significant improvement among the women who underwent TCA after one week of treatment, and the effects persisted up to 12 weeks after the last session. The differences were significant between the groups G1 and G2 and between G1 and G3 after 6 and 12 weeks of treatment (Figure 2). 

**Figure 3:**
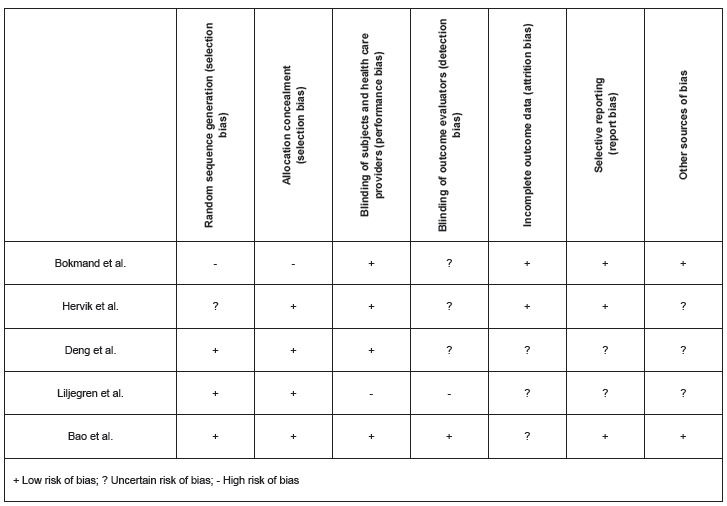
Summary of risk of bias, according to the selected study

The other two trials (Studies 3 and 5) found that both interventions (TCA and SA) were beneficial in the treatment of hot flashes. However, the differences between the groups in these studies were not significant (p > 0.05)^33^ ^,^ ^35^. Figure 3 presents the risk of bias analysis for the selected studies.

Regarding the risk of bias in the selected studies, it was observed that the reliability of the results can be questioned in all studies because of both uncertain risk of bias and high risk of bias. Uncertain risk of bias was prominent in the following areas: blinding of outcome evaluators (60%), incomplete outcome data (60%), other sources of bias (60%), and selective reporting (40%). High risk of bias was present in the following areas: random sequence generation (20%), allocation concealment (20%), blinding of subjects and health care providers (20%), and blinding of outcome evaluators (20%). In general, the areas where low risk of bias was prevalent were allocation concealment (80%), blinding of subjects and health care providers (80%), and random sequence generation (60%).

## Discussion

Based on the interventions used and the outcomes of the reviewed studies, we observed that acupuncture acts as a complementary therapy to conventional treatment for the control of hot flashes in menopausal women with breast cancer.

In the present review, three studies demonstrated that compared with SA, TCA was more effective for reducing the frequency and intensity of hot flashes(8,32,34). Other studies corroborate these findings, such as a pilot study conducted in South Korea that provided 12 TCA sessions for 10 post-menopausal women with breast cancer undergoing hormonal treatment with tamoxifen(r) or anastrozole(r). That study showed that the frequency of hot flashes was reduced from 9.3 to 1.5 daily episodes (p = 0.007) at the end of the treatment and that the intensity (measured using a VAS from 0 to 10) varied from 2.8 to 1.1 (p = 0.001), suggesting the effectiveness of TCA(36). 

Another study(37) examined the evidence supporting the effectiveness of TCA for hot flashes induced by menopause and found that TCA is an effective complementary therapy for reducing such symptoms. The same study noted that TCA may affect the release of serotonin and beta-endorphins in the central nervous system, therefore influencing and stabilizing the thermoregulatory center, normalizing body temperature, and reducing hot flashes and sweating(37). A systematic review conducted in 2011 presented a hypothesis for the emergence of hot flashes: dysfunction of the thermoregulation core, which keeps the body temperature within normal parameters (homeostasis). Women with complaints of hot flashes may have an alteration in this thermoregulation zone, which may be intensified by low levels of body estrogen that lead to dysregulated levels of noradrenaline and serotonin, which are involved in temperature regulation. This review reinforces suggestions that TCA may influence the release of serotonin and is a therapeutic modality for the relief of hot flashes(1). 

A recent review of the literature reported evidence that TCA improves immune function via the modulation of natural killer (NK) cell activity. A hypothetical model has been proposed to explain how TCA stimulates the immune system by stimulating the acupoint ST36. This point is also known as "immune enhancement acupoint" for its ability to improve immune system functioning. Stimulation of this acupoint induces the release of nitric oxide, a neurotransmitter that stimulates the lateral area of the hypothalamus via sensory nerves and promotes the secretion of opioid peptides, such as β-endorphin. This peptide reaches the spleen and other body areas through blood circulation, binding to the opioid receptors expressed on the surface of NK cells. By binding to these receptors, β-endorphin stimulates NK cells to increase the expression of cytotoxic molecules, which increases tumoricidal activity and, consequently, the production of IFN-γ. This cytokine induces the expression of NK cell receptors and possibly the secretion of cytokines from other immune system cells, orchestrating and amplifying anticancer immune functions(38).

In addition to promoting relief from hot flashes, TCA has been shown to be effective in relieving other symptoms in women with breast cancer. A study in the United States examined 51 women with breast cancer to evaluate the benefits of TCA for improving pain, stiffness, and functional capacity in women with arthralgia induced by treatment with aromatase inhibitors. The study compared TCA with SA and found that TCA was more effective than SA for reducing pain (p<0.001). The group undergoing TCA had a significantly (p = 0.003) lower mean pain intensity score (2.6) compared with the group undergoing SA (4.5) after six weeks of intervention(19).

Two other studies included in the present systematic review suggest that both interventions, i.e., TCA and SA, are beneficial for the treatment of hot flashes because there was a reduction in symptoms, even though there were no significant differences between the two intervention methods(33,35). However, studies suggest that there is controversy regarding the use of SA because the similarity between the results of TCA and SA may arise because the patient's expectations regarding the intervention influence its effects, or it could be related to the different administration protocols used. This is a crucial aspect of clinical research that requires rigorous evaluation, with protocols based on clinical guidelines(39-40).

One study(41)noted that TCA and SA are integral parts of health care and can have positive impacts on treatment outcomes. It also stated that the placebo effect of SA may be relevant because of various non-specific contextual factors that can arise during each treatment session(41). This premise is also supported by another study(42) that suggests that acupuncture therapy (both TCA and SA) involves an interpersonal and empathic relationship between the therapist and the patient, including stimulating verbal and non-verbal communication. The study also highlights the importance of the SA group in randomized trials, which should have study groups must be allocated identically except for the intervention under investigation because other differences could interfere with the study results(42).

Regarding the methods used to measure hot flashes, we found that most studies used diaries as a resource for recording the frequency and intensity of hot flashes. A study conducted to examine the methodological issues of hot flash assessments raised the importance of the validity and reliability of the measurement instruments(43). Regarding validity, the authors called for analyzing data pertaining to quality of life, treatment toxicity (appetite loss, nausea, insomnia), behavioral habits (physical activity) and treatment progression to eliminate measurement and confounding biases. One source of reliability is the use of a control group to evaluate the effectiveness of the intervention(43). In the present review, we found that only one study(35) evaluated other symptoms (such as quality of life, sleep disturbances, anxiety, depression, and symptoms of menopause) via validated scales with high reliability to diminish measurement and confounding biases. 

Regarding the long-term effects of TCA, a systematic review that aimed to assess these effects on the relief of hot flashes in women with breast cancer and men with prostate cancer after oncologic treatment showed that these effects lasted up to three months after the intervention with TCA. After treatment with TCA (from 5 to 12 weeks), an average reduction of 43.2% (general reduction for both men and women) in vasomotor symptoms was observed; and after three months, the reduction was maintained at 45.6%, demonstrating ongoing effectiveness after the end of the intervention(44).

Another study(45) followed 61 women with breast cancer two years after the end of treatment with TCA and SA and identified that the effect of the intervention was sustained in the TCA group compared with the SA group. However, the difference between the groups was not statistically significant. The authors also claimed that acupuncture may influence the thermoregulatory system by stimulating long-term neurotransmitters, thus providing an effective and safe alternative for treating hot flashes associated with menopause when performed by a qualified and competent professional(45).

### Study limitations

Most of the included studies had small sample sizes, were not representative of the population, and did not report on the sample size calculation, which may have compromised their ability to detect significant differences between the two interventions in relieving hot flashes. Thus, to minimize type I and type II errors and to increase the accuracy of the results, we suggest conducting new clinical trials with adequate sample sizes. In addition, most of studies did not report the loss of participants, especially in terms of follow up, which led to selection bias in these studies. Furthermore, the short follow-up time (4 to 12 weeks) may have affected the measurement of some outcomes. Therefore, we suggest that new RCTs be conducted with longer follow-up times. In addition to methodological limitations, the risk of bias found in the selected studies reduces the reliability of the findings, demonstrating the need for further large-scale studies with representative samples and a low risk of bias. 

## Conclusions

The results of this systematic review provide the following evidence regarding the effectiveness of TCA versus SA for the treatment/relief of hot flashes in menopausal women with breast cancer: 

- There is no sufficient evidence to affirm the effectiveness of TCA compared with SA for the treatment of hot flashes in menopausal women with breast cancer;

- It is possible to consider a slight superiority in the effectiveness of TCA compared with SA based on the studies reviewed. However, other well-designed clinical trials with long-term follow-up are needed to confirm this hypothesis; the groups undergoing both interventions should have a follow-up longer than 12 weeks to verify the long-term outcomes. 
